# Single-Cell RNA-seq Reveals Obesity-Induced Alterations in the *Brca1*-Mutated Mammary Gland Microenvironment

**DOI:** 10.3390/cancers12082235

**Published:** 2020-08-10

**Authors:** Pang-Kuo Lo, Yuan Yao, Qun Zhou

**Affiliations:** VA Maryland Health Care System, Department of Biochemistry and Molecular Biology, Greenebaum Cancer Center, University of Maryland School of Medicine, Baltimore, MD 21201, USA; pklo5321@gmail.com (P.-K.L.); yyao150@gmail.com (Y.Y.)

**Keywords:** obesity, breast cancer, high-fat diets, single-cell RNA sequencing (scRNA-seq), microenvironment, stroma, immune cells

## Abstract

Clinical and experimental studies have shown that obesity increases the development and progression of breast cancer. The impact of obesity on the tumor microenvironment plays an important role in tumorigenesis, yet the precise mechanisms underlying obesity-mediated effects on cell-to-cell communication within the tumor microenvironment have been difficult to define. In this study, we conducted single-cell RNA sequencing (scRNA-seq) studies to investigate the impact of high-fat diet (HFD)-induced obesity on transcriptomic landscapes of stromal and immune cells in mammary glands of *Brca1−/−; p53+/−* mice, an animal breast cancer model. Hierarchical clustering and gene pathway enrichment analyses of scRNA-seq data showed that five different subtypes of stromal fibroblasts existed in mouse *Brca1*-mutated mammary glands. HFD-induced obesity led to upregulated expression of extracellular matrix (ECM) genes (*Col3a1*, *Col6a3*, *Eln*, and *Sparc*) and downregulated expression of immunoregulatory genes (*Iigp1* and *Cxcl10*) in these stromal subtype cells. These findings, taken together, suggest that obesity alters the ECM composition and immune ecosystem through modulating the functionality of mammary stromal fibroblasts. Moreover, scRNA-seq analysis of mammary immune cells indicated that HFD-induced obesity promoted the generation and/or recruiting of pro-tumorigenic M2 macrophages in mammary glands. Our studies provide new insight into a mechanistic paradigm wherein obesity modulates the functions of stromal and immune cells to create the tumorigenic microenvironment for promoting breast tumorigenesis.

## 1. Introduction

Obesity has been recognized as a leading public health problem throughout the world [1]. Roughly two-thirds US people are overweight (Body Mass Index (BMI) ≥ 25 kg/m2) and over 50% of these overweight people are obese (BMI ≥ 30 kg/m2) [2]. An increasing number of large-cohort studies have demonstrated that obesity is susceptible to a risk of diseases, in particular cancer [1,2,3]. The large-scale epidemiological studies have also shown that obese women have a higher incidence to develop breast cancer and obese breast cancer patients tend to have the poor prognosis [4,5,6,7,8]. Multiple mechanisms have been revealed to account for the correlation between obesity and breast cancer, such as obesity-induced alterations in adipose endocrine functions, systemic immunity and metabolic homeostasis [9,10,11,12,13]. Nevertheless, the exact mechanisms underlying obesity-mediated breast cancer progression remain incomplete.

In addition to genetic and epigenetic aberrations in breast epithelial cells to drive their tumorigenesis, accumulating evidence has demonstrated that breast tumor onset and development are dramatically influenced by the surrounding microenvironment [14,15,16,17]. The tumor microenvironment is composed of multiple cell types (e.g., stromal cells, various innate and adaptive immune cells, and endothelial cells for forming the vasculature), metabolites, secreted growth factors, cytokines, angiogenic factors, and extracellular matrix (ECM) [18,19,20,21,22]. Given that obesity dysregulates the systemic metabolism of nutrients and the local availability of nutrient substrates in the tissue stroma, obesity has global and local impacts on endocrine actions, metabolism, inflammation and tissue homeostasis [23,24,25]. Due to the epidemic relevance of obesity in breast cancer development, investigating how obesity alters the local microenvironment in mammary glands is essential for understanding driving mechanisms underlying obesity-mediated breast cancer initiation, progression, and metastasis. Regarding the immunoregulatory effects of obesity, the majority of studies investigating the linkage between obesity and cancer have been aimed at the systemic inflammatory impacts of adiposity [26,27]. However, a characterization of obesity-induced inflammation and immune-related signaling pathways in the tumor microenvironment at single-cell resolution is not established.

Given that BRCA1 plays a crucial role in maintaining genomic stability through its function in DNA repair, *BRCA1* mutations facilitate breast and ovarian tumorigenesis via promoting genomic instability. Conditional *Brca1* knockout mice have been established to study the role of Brca1 in breast cancer development [28]. These mice developed mammary tumors at a low frequency (approximate 25%) after long latency (two years) [28], suggesting other factors are also involved in *Brca1*-mutation-dependent breast tumorigenesis. Indeed, introducing the *p53+/−* genetic alteration led to the early onset of mammary tumors in conditional *Brca1* knockout mice. Over 50% of *C57BL6 Brca1−/−; p53+/−* mice developed mammary tumors within 8 months of age [29]. Analysis of primary mammary tumors from *Brca1−/−; p53+/−* mice showed that multiple genetic/molecular alterations occurred during mammary tumorigenesis, including overexpression of ErbB2, c-Myc, p27 and Cyclin D1, and the loss of ERα and p16 expression [29]. As the characteristics of mammary tumors developed in *Brca1−/−; p53+/−* mice are significantly akin to human breast cancer, this animal tumor model is useful for studying breast cancer biology. Currently there are no studies addressing the impact of obesity on BRCA1-related breast cancer development. Therefore, *Brca1−/−; p53+/−* mice are an ideal animal model to address this question.

Single-cell RNA sequencing (scRNA-seq) approaches have provided the unprecedented detail of the cellular composition within the tumor microenvironment. Here we characterized the impacts of obesity on transcriptomic landscapes of stromal and immune cells within the mammary gland using scRNA-seq. Our scRNA-seq study focused on mammary glands of female *C57BL6 Brca1−/−;p53+/−* mice, the aforementioned animal model genetically predisposed to develop breast cancer [29]. We were interested in investigating the impact of obesity on the mammary gland microenvironment under the *Brca1−/−; p53+/−* background. High-fat diets (HFD) were used in the study to induce obesity in female *C57BL6 Brca1−/−; p53+/−* mice before they were able to develop mammary tumors, which allowed us to study the influence of obesity on the pre-malignant mammary gland microenvironment. The scRNA-seq analysis results revealed that HFD-induced obesity altered stromal transcriptomics to dysregulate expression of genes involved in modulating ECM homeostasis and immune signaling. Moreover, the scRNA-seq study also showed that HFD-induced obesity led to aberrations in the transcriptomic landscape of the mammary immune ecosystem to shift its functionality from pro-inflammation to anti-inflammation. Our findings, taken together, suggest that HFD-induced obesity chronically establishes a tumorigenic microenvironment to promote breast cancer development through the modifications of ECM homeostasis and the immune ecosystem in the *BRCA1*-mutated mammary gland.

## 2. Results

### 2.1. High-Fat Diets Induce Obesity in Brca1−/−; p53+/− Mice for scRNA-seq Analysis

**Figure 1:**
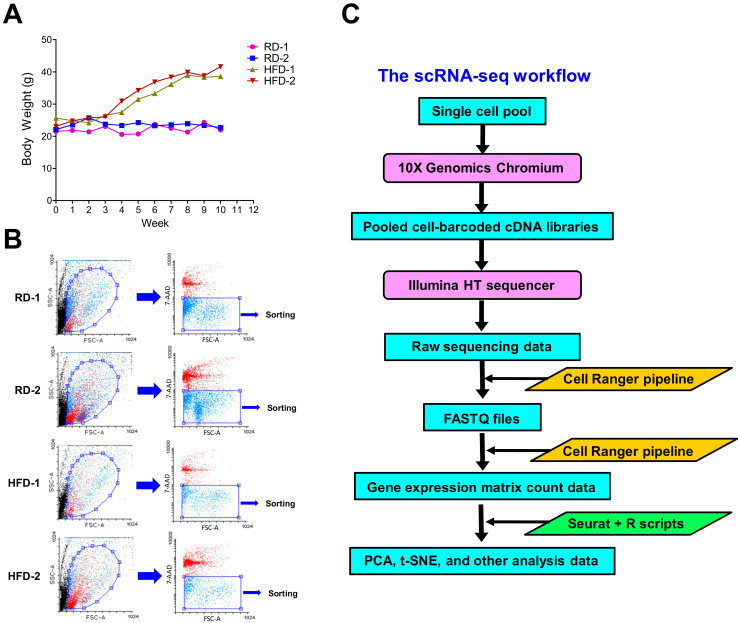
The scRNA-seq analysis of fluorescence-activated cell sorting (FACS)-sorted cells isolated from mammary glands of HFD or RD *Brca1−/−; p53+/−* mice. (**A**) Feeding with high-fat diets induced obesity in *Brca1−/−; p53+/−* mice. Body weight data of experimental mice were plotted against the week information. Four female *C57BL6 Brca1−/−; p53+/−* mice were involved in the experiment (the regular diet group: RD-1 and RD-2; the high-fat diet group: HFD-1 and HFD-2). (**B**) FACS sorting of viable mammary cells. Enriched non-epithelial cells were stained with 7-AAD dye and then sorted using the gated condition shown in the FACS data. 7-AAD-negative cells were gated for sorting viable cells. (**C**) The diagram of the scRNA-seq workflow.

To induce *Brca1−/−; p53+/−* mice to develop obesity, we fed two-month-old female mice with high-fat diets (HFD) for 10 weeks. Female mice with the similar age in the normal-weight group were fed with regular diets (RD) for the same period of time. During the diet-feeding period, we measured their weights weekly. Figure 1A shows that mice fed with high-fat diets significantly gained weight to average 40.11 ± 2.14 g after 10 weeks when compared to the control mouse group with average weight of 22.33 ± 0.52 g. At the end of diet-feeding experiments, experimental mice were euthanized and their mammary glands were harvested for single cell preparation as described in "Materials and Methods". In this study, we only focused on the impact of HFD-induced obesity on stromal fibroblasts and immune cells. To enrich these cell types, we used the anti-EpCAM antibody to remove mammary epithelial cells. The enriched non-epithelial cell fractions were stained with 7-AAD dye and then subjected to cell sorting to isolate viable single cells (Figure 1B). Given that dead cells were stained with 7-AAD, 7-AAD-negative cells were gated for cell sorting to purify viable single cells (Figure 1B). Sorted viable single cells were subjected to scRNA-seq analysis using the 10× Genomics Chromium system. 

As shown in Figure 1C, the technological design of the 10× Genomics Chromium system for scRNA-seq is droplet-based microfluidics with microchannels for generating aqueous-oil-emulsion droplets [30]. Each droplet is ideally composed of the reagent for reverse transcription (RT), a single cell and a bead conjugated with oligonucleotides. The oligonucleotide sequence has the universal adapter sequence designed for PCR amplification, the cell barcode sequence specific to each bead, the unique molecular identifier (UMI) sequence for sequencing read analysis and the oligo(dT) sequence for RT [30]. The reverse transcription process occurs in encapsulated droplets and their synthesized cDNAs are pooled together, PCR-amplified and then subjected to next-generation sequencing. 

### 2.2. The scRNA-Seq Analysis Identifies Various Molecular Subtypes of Mammary Stromal Fibroblasts in Brca1−/−; p53+/− mice

**Figure 2:**
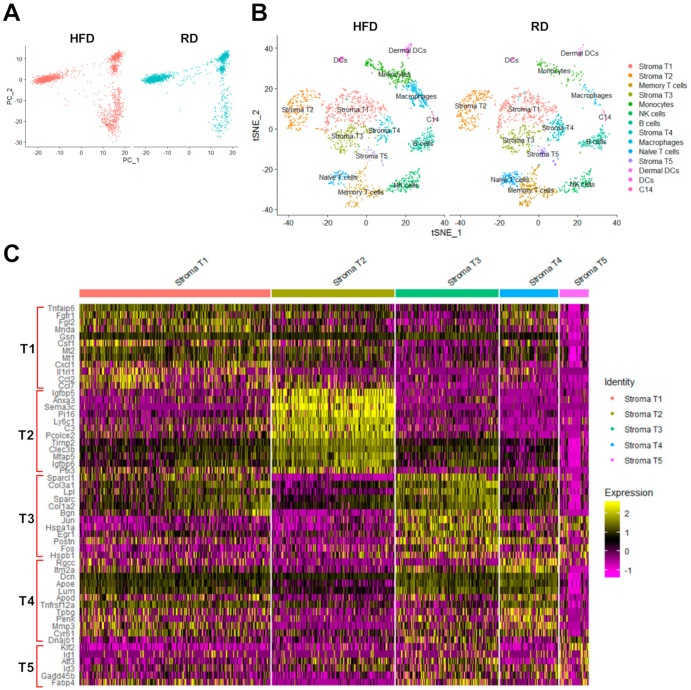
Dimensionality reduction and heatmap analyses of single-cell RNA sequencing (scRNA)-seq data. (**A**) PCA plot analysis of scRNA-seq data. (**B**) The t-SNE plot analysis of scRNA-seq data. Cell-type names are annotated in the plots based on identified cell-type-specific marker genes. (**C**) The heatmap of top differentially expressed genes in each stromal fibroblast subtype (Top 12 genes for T1–T4 and top 6 genes for T5).

Single-cell RNA sequencing read raw data were processed through the Cell Ranger analysis pipeline to generate scRNA-seq QC reports (Figures S1–S4) and three raw datasets (cell barcode, gene feature and count matrix) for each cell sample (Figure 1C) [31]. As shown in QC reports, over 600 cells per sample (RD-1: 1138, RD-2: 686, HFD-1: 1165, HFD-2: 903) were successfully sequenced (Figures S1–S4). The averages of total detected genes and median genes per cell for these four cell samples are 15,553 ± 309 and 1492 ± 257, respectively. The average percentage of reads mapped confidently to the mouse transcriptome is 66.05% ± 7.69%. We conducted scRNA-seq analysis of these three datasets (cell barcode, gene feature and count matrix) for each cell sample using the R-based bioconductor tool called Seurat (Figure 1C) [32,33]. As there were two scRNA-seq datasets for each diet group, we exploited Seurat to integrate them together before dimensionality reduction analysis [33]. Through analysis using dimensionality reduction algorithms built in Seurat, principal component analysis (PCA; Figure 2A) and t-distributed Stochastic Neighbor Embedding (t-SNE; Figure 2B) plots were generated to classify cell types (Figure 2). The t-SNE plots for HFD and RD groups show 14 different cell clusters (Figure 2B), indicating the presence of at least 14 distinct cell types in sorted cell samples. 

**Figure 3:**
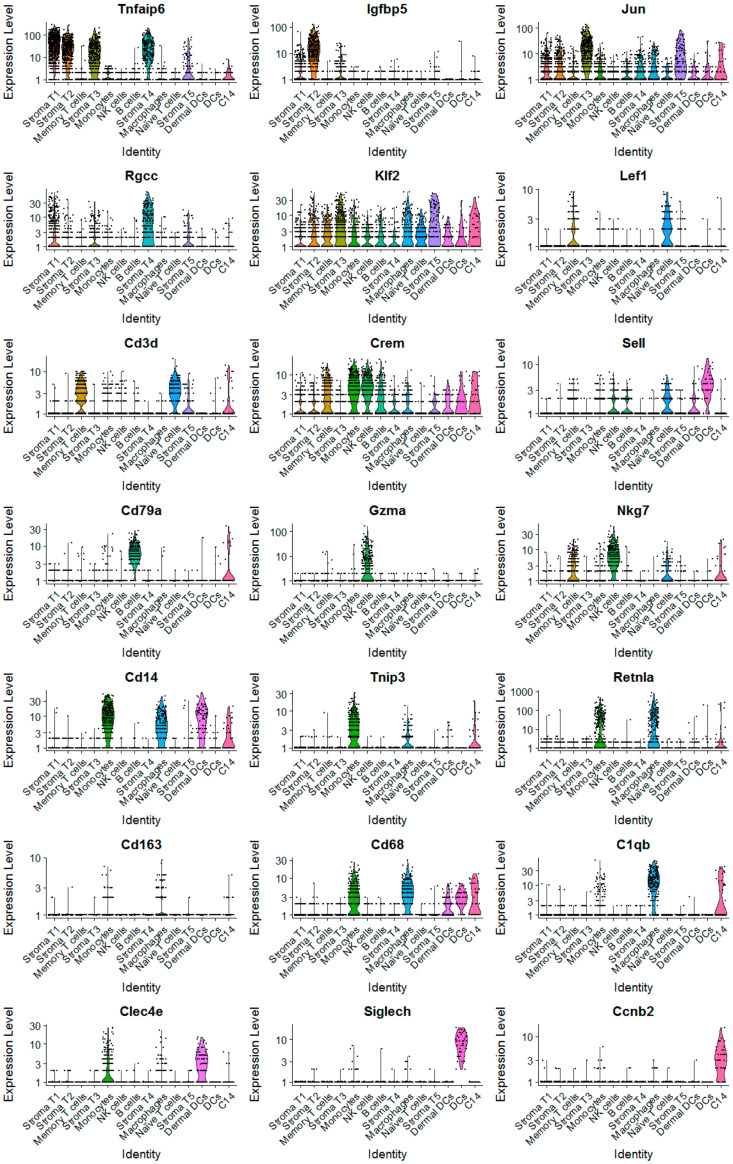
Expression analysis of cell-type-specific marker genes based on scRNA-seq data. Expression of these marker genes was presented in Vlnplots. The differential gene expression analysis algorithm built in the Seurat package was used to identify marker genes predominantly or uniquely expressed in each cell type.

**Figure 4:**
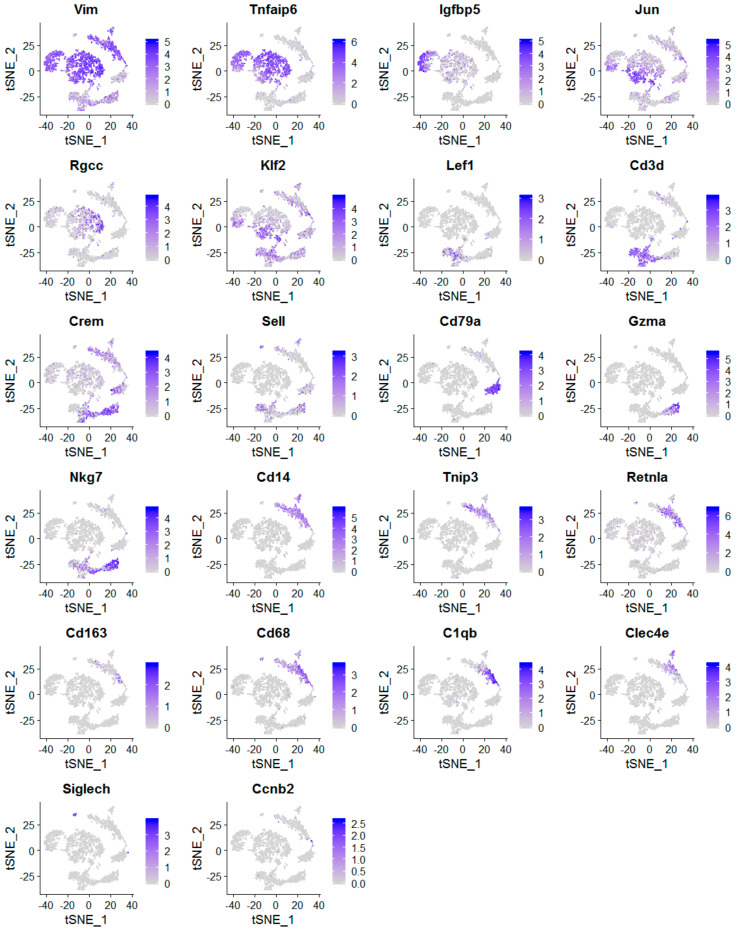
Expression profiles of cell-type-specific marker genes in mammary cell types. Gene expression profiles were presented in t-SNE plots.

Through analysis of individual cell cluster using the gene marker finder algorithm built in Seurat, we identified cell-type-specific gene markers for cell type classification. The expression of identified gene markers in these 14 cell clusters was displayed in Violin plots (Vlnplots; Figure 3) and gene-feature t-SNE plots (Figure 4). Based on these identified cell-type-specific gene markers shown in Figure 3 and Figure 4, we were able to assign known cell type names to 13 out of 14 cell clusters (Figure 2B), including stromal fibroblasts (Vim+; 5 different cell clusters), memory T cells (Cd3d+, Lef1+, Crem+), naive T cells (Cd3d+, Lef1+, Sell+), B cells (Cd79a+), NK cells (Gzma+, Nkg7+), moncytes (Cd14+, Tnip3+, Retnla+, Cd68+), macrophages (Cd14+, Retnla+, Cd68+, Cd163+, C1qb+), dermal dendritic cells (dermal DCs; Cd14+, Clec4e+) [34] and dendritic cells (DCs; Siglech+) [35]. Through scRNA-seq analysis, we for the first time identified five different types of stromal fibroblasts present in *Brca1−/−; p53+/−* mammary glands, including stroma T1 (Tnfaip6^high^), stroma T2 (Igfbp5+), stroma T3 (Jun^high^), stroma T4 (Rgcc+) and stroma T5 (Klf2^high^) (Figure 2, Figure 3 and Figure 4). 

**Figure 5:**
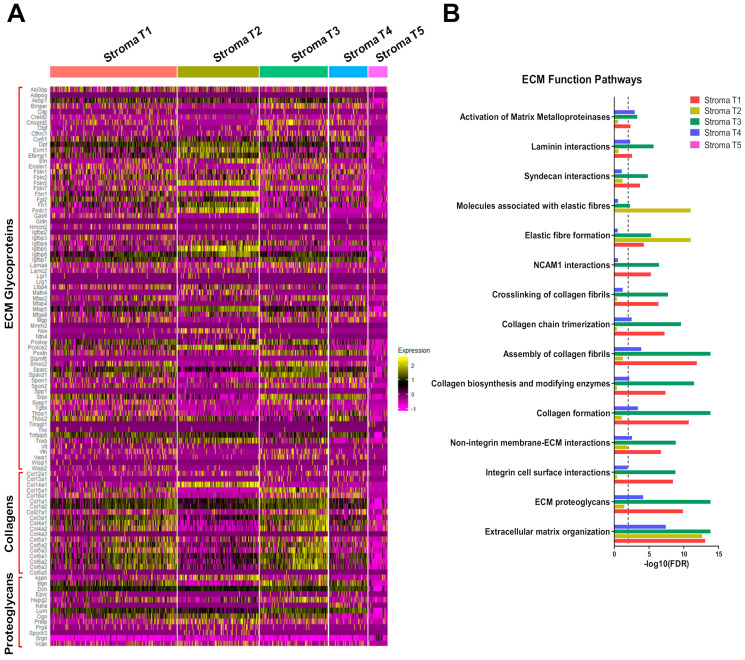
Expression profiling and gene pathway enrichment analyses reveal common and distinct functional roles of mammary stromal fibroblast subtypes in the ECM. (**A**) Expression profiles of matrisome genes in five stromal fibroblast subtypes. Genes encoding ECM core components including glycoproteins, collagens and proteoglycans were analyzed and shown in the heatmap. (**B**) Enrichment analysis of differentially expressed genes in mammary stromal fibroblast subtypes in ECM function pathways. The FDR cut-off value (0.01, −log10(FDR) = 2) to judge the statistical significance of the enrichment is depicted as a dash line in the graph.

Through the gene marker finder algorithm built in Seurat, we identified differentially expressed genes that specifically stratify stromal fibroblasts into five subtypes. The hierarchical clustering analysis of top differentially expressed genes of each stromal fibroblast subtype is shown as a heatmap (Figure 2C). Given that biosynthesis and modification of the extracellular matrix (ECM) are major functions of stromal fibroblasts, we also performed expression profiling analysis of genes encoding ECM-related genes included in the matrisome gene set [36] using our stromal fibroblast datasets. As shown in the heatmap data (Figure 5A), these identified stromal fibroblast subtypes displayed some common and unique expression patterns of matrisome genes, except the T5 subtype that lacked expression of most matrisome genes. Therefore, this analysis suggests that stromal fibroblast subtypes T1 to T4 have their common and unique functions in the ECM. For example, expression of genes encoding collagens and enzymes for collagen biosynthesis and modification was significantly enriched in stromal fibroblast subtypes T1 and T3, indicating that these two fibroblast subtypes have specific functions in collagen formation and modification. The stromal fibroblast subtype T2 predominantly expressed nine ECM-related genes (*Ecm1*—extracellular matrix protein 1, *Fbln5*—fibulin 5, *Fbn1*—fibrillin 1, *Fndc1*—fibronectin type III domain containing 1, *Igfbp5*—insulin like growth factor binding protein 5, *Nov/Ccn3*—cellular communication network factor 3, *Pcolce2*—procollagen C-endopeptidase enhancer 2, *Col14a1*—collagen type XIV alpha 1, *Aspn*—asporin) when compared to other stromal fibroblast subtypes (Figure 5A), suggesting that this fibroblast subtype plays a key role in supplying these ECM components and regulatory factors in mammary glands. 

To determine the biological functions of these five distinct stromal fibroblasts, we performed gene pathway enrichment analysis of differentially expressed genes in these five fibroblast subtypes using the Reactome [37]. We selected differentially expressed genes based on the criteria of log2(fold change) > 0.5 and adjusted p value (FDR) < 0.001. We detected 249, 230, 249, 169 and 34 differentially expressed genes in fibroblast subtype T1, T2, T3, T4 and T5, respectively, which were used in gene enrichment analysis. Consistent with the heatmap data (Figure 5A), stromal fibroblast subtypes T1 and T3 specifically manifested gene signatures involved in collagen formation, collagen biosynthesis and modifying enzymes, assembly of collagen fibrils, collagen chain trimerization, crosslinking of collagen fibrils and neural cell adhesion molecule 1 (NCAM1) interactions (Figure 5B). In contrast, the stromal fibroblast T2 predominantly expressed genes implicated in elastic fiber formation and molecules associated with elastic fibers (Figure 5B). Noticeably, the stromal fibroblast T5 lacked all of gene signatures for these ECM function pathways, suggesting that this fibroblast subtype has no significant role in the ECM organization (Figure 5B). Moreover, we also identified other biological pathways differentially enriched in these stromal fibroblast subtypes. As shown in Figure S5A, differentially expressed genes in the stromal fibroblast T1 were enriched in multiple immune-related pathways, including immune-related signaling pathways by interleukins and cytokines, implying that this fibroblast subtype plays a crucial role in regulating the immune system in mammary glands. Particularly, the stromal fibroblast T5 displayed unique gene expression signatures enriched in Transforming growth factor β (TGFβ) and mitogen-activated protein (MAP) kinase pathways when compared to other fibroblast subtypes (Figure S5B). In addition, gene ontology enrichment analysis using Gorilla, [38] also showed that the stromal fibroblast T5 uniquely expressed transcription factor genes with functions involved in stem cell regulation and kinase signaling activity (FDR = 0.03), including *Nr4a1*—nuclear receptor subfamily 4, *Atf3*—activating transcription factor 3, *Klf2*—kruppel-like factor 2, *Klf6*—kruppel-like factor 6, *Jun*—jun oncogene, *Fos*—fbj osteosarcoma oncogene). These analyses suggest that the stromal fibroblast T5 is undifferentiated and has stem-cell properties. Intriguingly, both stromal fibroblast subtypes T3 and T5 predominantly expressed genes enriched in stress response pathways (Figure S5C), suggesting that these two fibroblast subtypes may play critical roles in response to tissue stresses and external stimuli. Our expression profiling and gene pathway/ontology enrichment analyses, taken together, demonstrate that these identified stromal fibroblast subtypes share some similar characteristics and also possess their unique functional properties.

### 2.3. Obesity Aberrantly Modulates the Transcriptomic Landscape of Brca1−/−; p53+/− Mammary Stromal Fibroblasts to Alter their Functions in the Extracellular Matrix and Immunological Regulation

**Figure 6:**
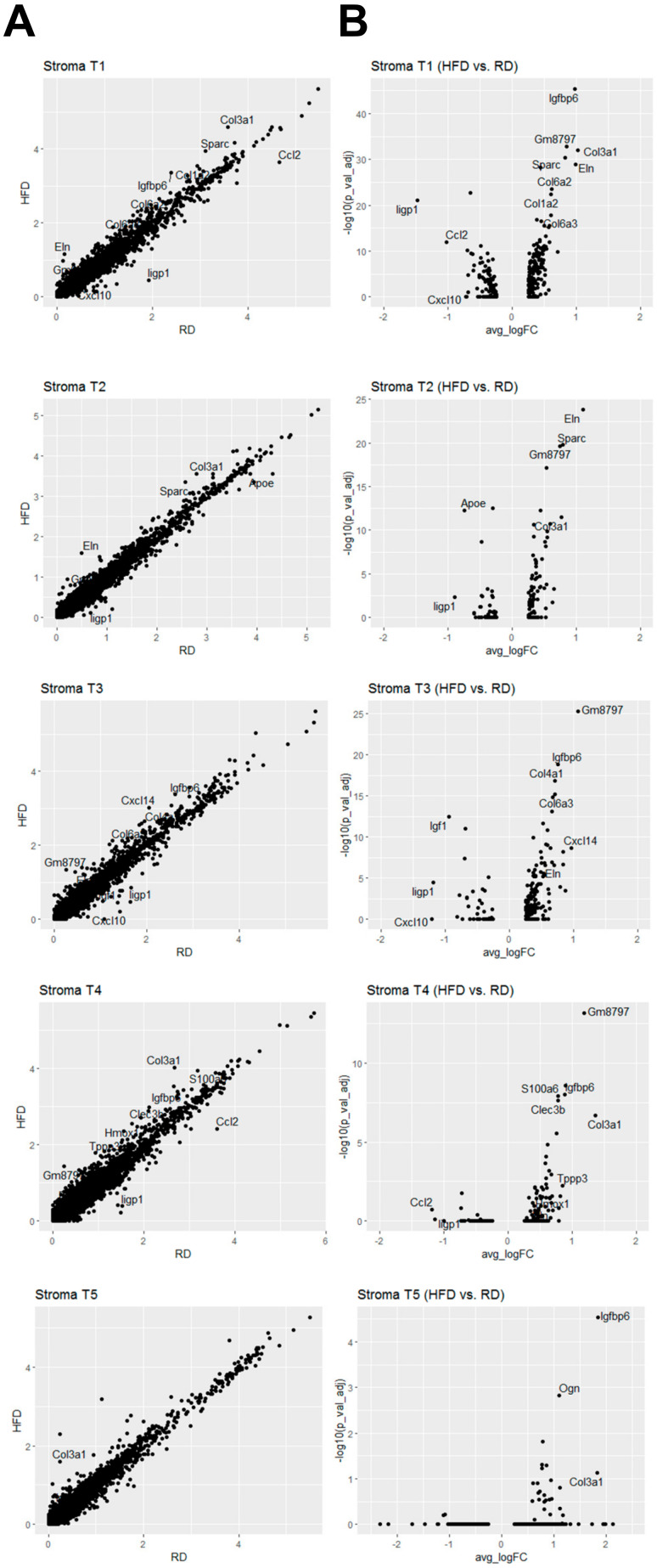
Differential gene expression analysis of HFD stromal cell subtypes in comparison with RD counterparts. (**A**) Scatter plot analysis of differentially expressed genes in HFD vs. RD stromal cell subtypes. (**B**) Volcano plot analysis of differentially expressed genes in HFD vs. RD stromal cell subtypes.

**Figure 7:**
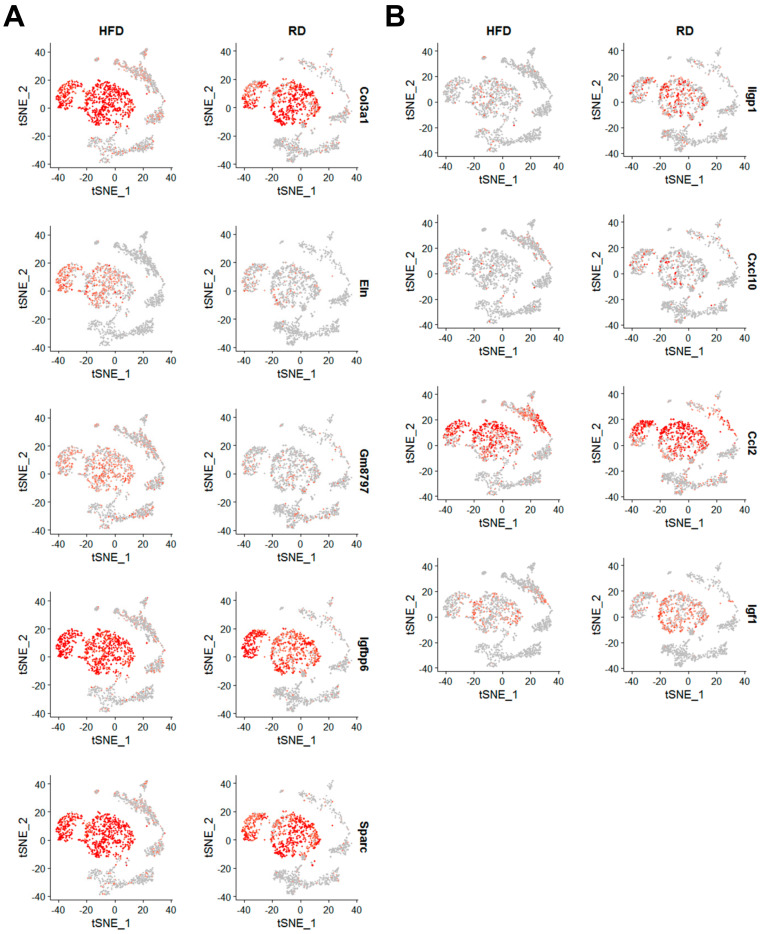
Expression profiles of HFD-induced differentially expressed genes in mammary stromal cell subtypes. (**A**) Expression patterns of HFD-induced upregulated genes were displayed in t-SNE plots. (**B**) Expression patterns of HFD-induced downregulated genes were displayed in t-SNE plots.

According to these classified cell types (Figure 2B), we performed differential gene expression analysis of each cell type across these two diet conditions. Differentially expressed genes in these stromal cell types (HFD vs. RD) were displayed in scatter (Figure 6A) and volcano (Figure 6B) plots. Although scatter and volcano plots both show what genes are differentially expressed, volcano plots provide the additional information about adjusted p values for determining whether differentially expressed genes are statistically significant. We set cut-off values (≥1.5-fold, adjusted *p*-value < 0.05) to identify differentially expressed genes. Expression of 13 genes were upregulated in HFD mammary stromal cell types when compared to RD counterparts, including Clec3b (in Stroma T4), Col1a2 (in Stroma T1), Col3a1 (in Stroma T1, T2, T4, T5), Col4a1 (in Stroma T3), Col6a2 (in Stroma T1), Col6a3 (in Stroma T1, T3), Cxcl14 (in Stroma T3), Eln (in Stroma T1, T2, T3), Gm8797 (in Stroma T1, T2, T3, T4), Igfbp6 (in Stroma T1, T3, T4, T5), Ogn (in Stroma T5), S100a6 (in Stroma T4), and Sparc (in Stroma T1, T2) (Figure 6). Among these upregulated genes, Col3a1, Col6a3, Eln, Gm8797, Igfbp6 and Sparc were detected in two or more mammary stromal cell types (Figure 6B and Figure 7A). Both Col3a1 and Col6a3 encode collagens that are extracellular matrix proteins. Eln encodes elastin that is also an extracellular matrix protein. Sparc encodes a secreted acidic cysteine rich glycoprotein that is involved in extracellular matrix synthesis and the regulation of cell shape. Gm8797 is the mouse ubiquitin B pseudogene. Igfbp6 encodes insulin-like growth factor binding protein 6 that is implicated in regulation of insulin-like growth factor signaling. Given that four out of six upregulated genes are related to the extracellular matrix (ECM), our findings suggest that HFD-induced obesity alters the functionality of mammary stromal fibroblasts to change the composition of the ECM in mammary glands of Brca1−/−; p53+/− mice.

In addition to these upregulated genes, we also indentified five downregulated genes in HFD mammary stromal cell types, including *Apoe* (in Stroma T2), *Ccl2* (in Stroma T1), *Cxcl10* (in Stroma T1, T3), *Igf1* (in Stroma T3), and *Iigp1* (in Stroma T1, T2, T3) (Figure 6 and Figure 7B). Among these downregulated genes, *Iigp1* expression was significantly downregulated in most of mammary stromal cell types (Figure 7B). *Iigp1* encodes interferon inducible GTPase 1 whose expression is induced by interferons [39]. Iigp1 is implicated in cell-autonomous resistance to intracellular pathogens via the autophagy mechanism [40].

Two other immunity-related chemokine genes, *Ccl2* and *Cxcl10*, encode C-C motif chemokine ligand 2 and C-X-C motif chemokine ligand 10, respectively. Ccl2 is implicated in immunoregulatory and inflammatory processes through its chemotactic activity for monocytes and basophils [41]. The chemokine activity of Ccl2 is mediated by its binding to chemokine receptors CCR2 and CCR4. Obesity has been shown to induce Ccl2 expression in mouse mammary glands for promoting macrophage recruitment and angiogenesis, suggesting that Ccl2 is a pro-tumorigenic, invasive factor for breast cancer [42,43]. This discrepancy might have been due to differences in animal models and experimental approaches between our and other studies. Further studies are necessary to clarify this discrepancy. Through binding to the CXCR3 receptor, Cxcl10 has multiple regulatory roles in immunity and the ECM, including stimulation of monocytes, natural killer and T-cell migration, and modulation of adhesion molecule expression [44]. *Igf1* encodes insulin-like growth factor 1 that is involved in regulating cell growth and metabolism. *Apoe* encodes apolipoprotein E that is involved in catabolism of triglyceride-rich lipoprotein constituents. Given that the mainly prominent downregulated genes (*Iigp1* and *Cxcl10*) play critical roles in immunoregulation and inflammation, these findings suggest that HFD-induced obesity suppresses the ability of mammary stromal fibroblasts to express and secrete these immunoregulatory and inflammatory proteins/cytokines to compromise the immunity of mammary glands.

### 2.4. Obesity Aberrantly Regulates the Immune Ecosystem within Brca1−/−; p53+/− Mammary Glands to Establish a Tumor-Favorable Tissue Microenvironment

**Figure 8:**
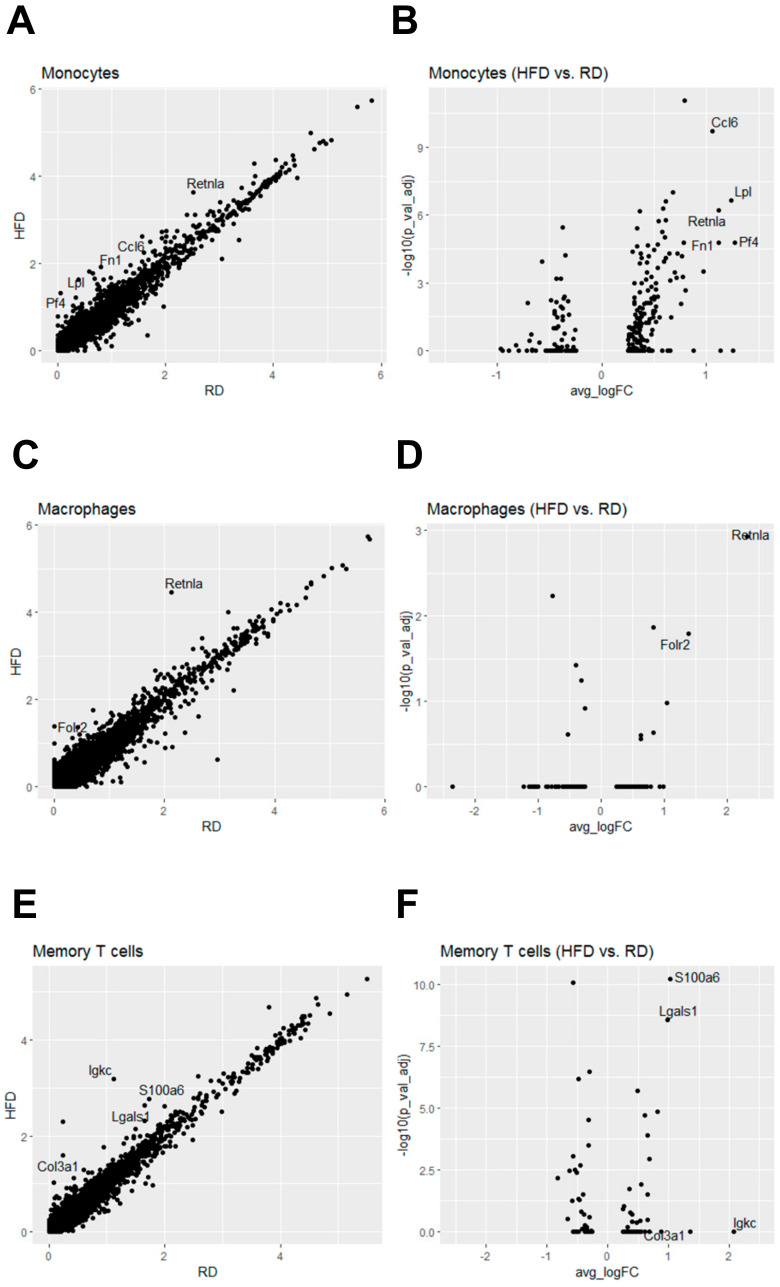
Differential gene expression analysis of HFD monocytes, macrophages and memory T cells in comparison with their respective RD counterparts. (**A**) Scatter plot analysis of differentially expressed genes in HFD vs. RD monocytes. (**B**) Volcano plot analysis of differentially expressed genes in HFD vs. RD monocytes. (**C**) Scatter plot analysis of differentially expressed genes in HFD vs. RD macrophages. (**D**) Volcano plot analysis of differentially expressed genes in HFD vs. RD macrophages. (**E**) Scatter plot analysis of differentially expressed genes in HFD vs. RD memory T cells. (**F**) Volcano plot analysis of differentially expressed genes in HFD vs. RD memory T cells.

**Figure 9:**
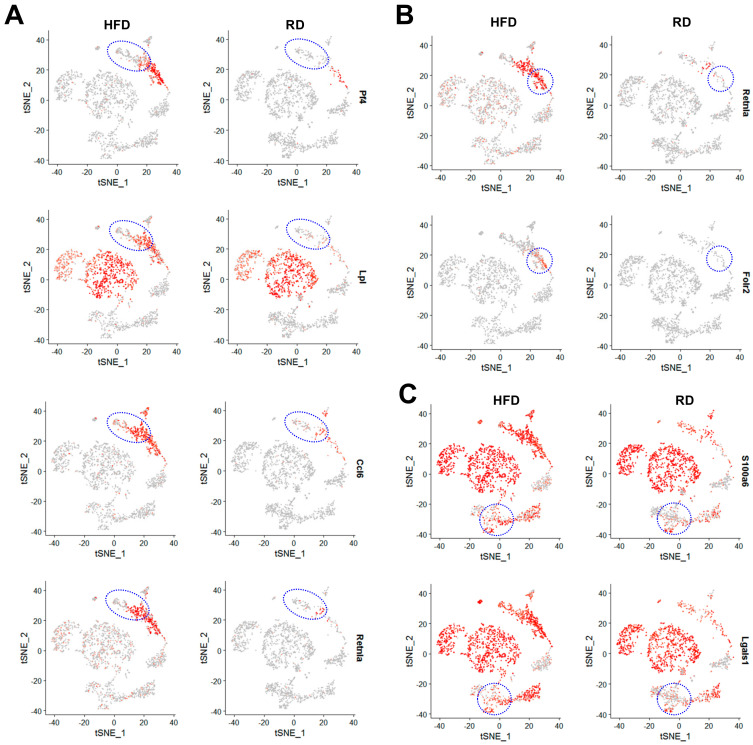
Expression profiles of HFD-induced differentially expressed genes in mammary monocytes, macrophages and memory T cells. Expression patterns of upregulated genes in HFD monocytes (**A**), HFD macrophages (**B**) and HFD memory T cells (**C**) were displayed in t-SNE plots.

To reveal the impact of HFD-induced obesity on transcriptomic profiles of immune cells, we also conducted differential gene expression analysis on immune cell types. Among these eight immune cell types, we identified differentially expressed genes in monocytes, macrophages and memory T cells using the cut-off criterion (≥2-fold, adjusted *p*-value < 0.05). In monocytes, we identified that expression of five genes was significantly upregulated by HFD-induced obesity, including *Ccl6*, *Fn1*, *Lpl*, *Pf4* and *Retnla* (Figure 8A,B and Figure 9A). In macrophages, expression of *Retnla* along with another gene *Folr2* was upregulated by HFD (Figure 8C,D and Figure 9B). In memory T cells, HFD led to upregulated expression of two genes *Lgals1* and *S100a6* (Figure 8E,F and Figure 9C).

Among these upregulated genes in immune cells, *Retnla/Fizz1* and *Folr2* are marker genes of M2 macrophages [45,46]. It has been shown that the polarization of M1 macrophages to M2 macrophages is the shift of macrophage functionality from a pro-inflammatory role to an anti-inflammatory role [47,48]. Given that tumor-associated macrophages (TAMs) known to promote tumorigenesis and metastasis manifest the M2-like phenotype, the anti-inflammatory function of M2 macrophages is pro-tumorigenic [49,50]. Upregulated expression of *Retnla/Fizz1* and *Folr2* genes in HFD macrophages (Figure 8C,D and Figure 9B) suggests that HFD-induced obesity stimulates the generation and/or recruiting of M2 macrophages in mammary glands, which is a sign of immunosuppression. *Retnla* encodes resistin-like molecule alpha with activity to suppress immunity [51] and promote tissue fibrogenesis via activation of myofibroblast differentiation [52]. *Folr2* encodes folate Receptor β whose expression is induced in M2-like TAMs [46]. In monocytes, HFD-induced obesity also led to upregulated expression of the M2 marker gene *Retnla/Fizz1* (Figure 8A,B and Figure 9A). Moreover, expression of the *Ccl6* gene, encoding chemokine (C-C motif) ligand 6, was also upregulated in HFD monocytes (Figure 8A,B and Figure 9A). *Ccl6* is also a M2 marker gene for macrophages as Ccl6 expression is induced in IL-13-treated macrophages with M2 polarization [53]. Upregulated expression of M2 marker genes in HFD monocytes suggests that HFD-induced obesity promotes differentiation of monocytes to M2 macrophages, consistent with the finding that HFD macrophages manifested the M2 gene signature profile (Figure 8C,D and Figure 9B).

In addition to M2 marker genes, HFD monocytes expressed higher levels of Pf4/Cxcl4 than RD monocytes (Figure 8A,B and Figure 9A). CXCL4 is known to have anti-immunity activity through its inhibitory effect on cytotoxic T lymphocytes [54]. Therefore, this finding suggests that HFD-induced obesity can trigger monocytes to express more Cxcl4 to suppress the function of cytotoxic T lymphocytes in mammary glands. Besides these chemokine genes, HFD also upregulated expression of *Lpl* (lipoprotein lipase) and *Fn1* (fibronectin 1) genes involved in regulation of lipoprotein/lipid metabolism and cell migration, respectively. 

In memory T cells, expression of *Lgals1* and *S100a6* genes was upregulated by HFD-induced obesity. *Lgals1* encodes galectin-1 that belongs to a family of β-galactoside-binding proteins involved in regulating cell-cell and cell-matrix interactions. *Lgals1* is a HIF-1-regulated gene and plays crucial pro-tumorigenic functions within the tumor microenvironment. Galectin-1 is known to enhance tumor angiogenesis through inhibiting T cell-mediated cytotoxic immune responses [55]. Based on these known roles of galectin-1, HFD-induced upregulation of Lgals1 expression in memory T cells may have an immunosuppressive effect. *S100a6*, encoding S100 calcium binding protein A6, is associated with poor prognosis of DCIS patients and several cancer types overexpress this *S100* gene, suggesting *S100a6* plays an oncogenic role in tumorigenesis [56]. S100a6 can be secreted from several tumor cell types, and has been implicated in regulation of cell cycle and CXCL14, a pro-inflammatory chemokine [57]. Although the functional role of S100a6 in T cells is unclear, HFD may trigger memory T cells to express and secrete more S100a6 to promote the tumorigenic process in mammary glands.

## 3. Discussion

Revealing the interactions between tumor cells and their surrounding microenvironment at single-cell resolution is critical to understand tumorigenic and metastatic processes. In this study, we exploited scRNA-seq to investigate the impact of obesity on the microenvironment in the mammary gland of a mouse Brca1-deficiency cancer model. Our study for the first time reveals that the mouse *Brca1*-mutated mammary stroma contains at least five different molecular subtypes of stromal fibroblasts with their respectively unique functional characteristics. Importantly, this study provides novel insight into how HFD-induced obesity deregulates the ECM homeostasis and immunity in the mammary gland of this animal mammary tumor model through altering the transcriptomic landscapes of stromal fibroblasts and some immune cell types.

**Figure 10:**
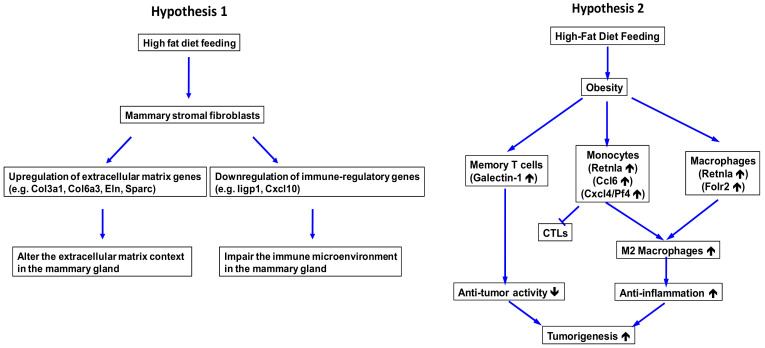
Two hypothetical models illustrating how HFD-induced obesity influences the mammary tissue microenvironment to promote breast tumorigenesis.

Through scRNA-seq analysis of HFD stromal fibroblasts relative to RD counterparts, we found that obesity promoted stromal fibroblasts to express ECM proteins (e.g., collagen, elastin, and glycoprotein). This finding suggests that these obesity-induced ECM proteins may lead to the increased fibrotic property of mammary adipose tissue, resulting in mammary gland tissue becoming dense and rigid. It has been reported that obesity causes the fibrotic remodeling of adipose tissue [58]. The obesity-induced mammary fibrosis has correlated with cancer incidence [59]. Further clinical studies employing mammography and palpation, have demonstrated that increased density and rigidity of breast tissue due to alterations in ECM are risk factors for breast cancer onset and progression [60,61]. Given that changes in ECM composition can dramatically affect cellular mechanosignaling, breast tissue stiffness (e.g., induced by obesity and tumorigenesis) enhances mechanosignaling to promote tumorigenesis of breast epithelial cells and their aggressive features through dysregulated growth factor and cytokine signaling [62,63,64,65,66]. Our scRNA-seq study is in line with these previous studies and suggests that obesity induces fibrotic remodeling in the mammary stroma to establish a pro-tumorigenic microenvironment for breast cancer development through altering local ECM mechanical properties and mechanotransduction (Figure 10, hypothesis 1). Moreover, we also found that obesity led to downregulated expression of immune-related cytokine genes in mammary stromal fibroblasts. Our data indicate that obesity promotes the interaction between immune cells and mammary fibroblasts, which may in turn foster breast cancer development (Figure 10, hypothesis 1).

In addition to the metabolic impacts on cancer initiation and promotion, obesity has been shown to aberrantly modulate immune homeostasis for changing the elimination, equilibrium, and escape phases of global as well as local immune-editing processes [67,68,69]. Moreover, multiple lines of studies have demonstrated that obesity drives various inflammatory alterations including the dysregulated functionality of immune cells, chronic changes in production of cytokines, gradual degeneration of both adaptive and innate immune systems and enhancing the immune aging process [67,69,70,71]. Nevertheless, the effect of obesity on the local immunity in the mammary gland remains elusive. To address this local impact, we also conducted scRNA-seq analysis of the transcriptomic profiles of HFD immune cells relative to RD counterparts. The most prominent finding from our study is that HFD-induced obesity significantly enhanced expression of genes related to the M2-type macrophage in monocytes and macrophages. This suggests that obesity facilitates the generation and/or recruiting of M2 macrophages in the mammary gland. Macrophages are a type of leukocytes functioning in the innate immune system and they are localized in almost all tissues. Macrophages play vital roles in anti-infective immunity through their ability in engulfing as well as digesting foreign pathogens and sustaining tissue homeostasis [72,73]. Macrophages are generated from differentiation of monocytes. Monocytes, derived from progenitor cells in the bone marrow, circulate in the blood stream and later are recruited to local tissues for differentiation into macrophages after stimulated by local microenvironmental factors [72,73,74,75]. Based on tissue-environmental conditions, macrophages can be polarized into two main phenotypes: M1 and M2 macrophages [47,48]. M1-type macrophages have pro-inflammatory and tumoricidal activity, whereas M2-type macrophages are anti-inflammatory as well as pro-angiogenic and play a pivotal role in wound healing through their effect to suppress inflammatory responses and enhance connective tissue remodeling [47,48]. Studies have shown that macrophages are recruited to the tumor microenvironment. These tumor-associated macrophages (TAMs) mainly display the M2-like phenotype [49,50]. Recent evidence suggests that TAMs play a critical role in growth, progression, and metastasis of various cancers, including breast cancer, due to their pro-tumorigenic activity [49,50,76]. Moreover, M2 macrophages have been found to participate in lung and liver fibrogenesis [77,78], suggesting another macrophage-mediated function to promote tumorigenesis. Notably, our novel findings support that obesity suppresses the immunity and promotes fibrogenesis in mammary gland tissue through enhancing development and/or recruiting of M2-type macrophages. Our findings solidify and reinforce our hypothesis that M2 macrophages are the potential mechanism for obesity to promote tumorigenesis of mammary epithelial cells (Figure 10, hypothesis 2). We were aware that obesity has been known to promote the recruitment of M1 macrophages to adipose tissue [79], which is different from our finding. However, as the *in vivo* plasticity of macrophage phenotypes is dramatically affected by the tissue microenvironment, the difference in our finding was potentially caused by the alterations in the *Brca1*-mutated mammary gland microenvironment. In addition to the dysregulation of monocytes and macrophages, obesity also compromises the immune function of memory T cells through upregulation of galectin-1 that functions to inhibit T-cell-mediated immunity and promote tumor angiogenesis (Figure 10, hypothesis 2). 

Prior single-cell RNA-sequencing studies have documented that normal mammary epithelial cells exhibit the genomic diversity and contain a CDH5 cell subpopulation as mammary stem cells [80]. Single-cell RNA-sequencing of primary triple-negative breast cancer (TNBC) cells identified the major tumor-derived signature among all TNBC cells is the Basal-Like 1 proliferative signature [81]. These observations, along with the immune and cancer-associated fibroblasts single-cell RNA-seq datasets reveal that single-cell transcriptome profiling can identify and characterize clinically important subpopulations [82,83]. However, no study to date has studied the impact of the high-fat diet on premalignant mammary microenvironment in *Brca1−/−; p53+/−* mouse models. Considering the diverse stromal cells, single-cell expression profiling is particularly important for the accurate characterization of mammary stromal cells in the high fat condition. To our knowledge our present study is the first report that comprehensively compared stromal heterogeneity and demonstrates the characteristics of different stromal cell subtypes that were shaped by the high fat diet in the mammary gland microenvironment. A limitation of our study is that we only studied stromal cells at one timepoint. It is possible that stromal cell populations can undergo changes as these stromal cells communicate with mammary epithelial cells at different timepoints. Future studies should aim to compare stromal cell types and stages of mammary premalignant (or malignant) epithelial cells to further elucidate high-fat-diet contributions to TNBC tumor development.

## 4. Materials and Methods

### 4.1. HFD-Induced Obesity in Brca1−/−; p53+/− Mice

Four two-month-old female *C57BL/6 Brca1*−/−; *p53+/*− mice were randomized and fed with either a lean-fat diet (10% kcal from fat) or a high-fat diet (60% kcal from fat) ad libitum for 10 weeks (*n* = 2 for each diet group). Both lean-fat (D12450J) and high-fat (D12492) diets were obtained from Research Diets (New Brunswick, NJ, USA). The body weights of experimental mice were measured once per week. After 10-week diet feeding, mice were euthanized and dissected, and then their mammary glands were taken for single cell isolation. This animal experiment was performed in accordance with federal guidelines and the animal use protocol (AUP # 0318023) approved by the Institutional Animal Care and Use Committee (IACUC) of the University of Maryland School of Medicine.

### 4.2. Single-Cell Isolation of Mammary Stromal and Immune Cells

For single cell isolation from mammary glands, dissected mammary glands were minced to the size of approximate 1 mm^3^ in the DMEM/F12 digestion medium containing 300 units/mL collagenase, 100 units/mL hyaluronidase, 10%FBS, and 1% antibiotics (Penicillin/Streptomycin). Minced mammary tissue was digested at 37 °C. The tissue digestion time depended on the tissue amount. After pipetting to dissociate mammary cells and centrifugation, tissue cells were treated with 5mg/mL dispase II and 0.1mg/mL DNase I in the DMEM/F12 medium at 37 °C for 5 minutes. After centrifugation, tissue cells were resuspended in 0.25% trypsin/EDTA for digestion at 37 °C for 1 minute. The cell mixture was mixed with 5 ml DMEM/F12 media with 10% FBS to neutralize trypsin. After tissue cells were spun down, they were resuspended in the red blood cell lysis buffer (00-4333-57, Ebioscience, San Diego, CA, USA) for 3 minutes at RT to lyse red blood cells. After centrifugation and the removal of the supernatant, tissue cells were washed twice with PBS containing 2% FBS. After PBS washing, resuspended cells were filtered through the 40 µm cell strainer. Non-epithelial cells were further enriched by removing epithelial cells using the anti-EpCAM antibody and magnetic dynabeads. To isolate viable cells, enriched non-epithelial cells were stained with 7-AAD dye (0.5 µg/mL) and 7-AAD-negative viable cells were sorted using the FACS Aria II cell sorter (BD Biosciences, Franklin Lakes, NJ, USA). 

### 4.3. Single-Cell RNA Sequencing and Data Analysis

FACS-sorted viable single cells were resuspended in PBS with 2% FBS and 2000 cells were used to make pooled single-cell cDNA libraries using 10× Genomics Chromium (Pleasanton, CA, USA). A TC20 Automated Cell Counter (BioRad, Hercules, CA, USA) was exploited to determine cell number and viability of sorted single cells. Only cell samples with at least 80% viability were processed for scRNA-seq analysis. We loaded cell samples into the 10× Genomics Chromium device to process them into scRNA-seq libraries according to the manufacturer’s protocol. Pooled scRNA-seq libraries were sequenced with a depth of 50,000–100,000 reads per cell using a Hiseq 4000 sequencer (Illumina, San Diego, CA, USA). The 10× Genomics Cell Ranger Analysis Pipeline (Version 3.0.2, 10x Genomics, Pleasanton, CA, USA) was used to process raw sequencing data for the generation of FASTQ files and the expression matrix counts of mouse genes via the alignment of sequencing reads to the mouse reference genome (mm10). We used the R-based Seurat package (Version 3.1.1) and R scripts (R version 3.6.1) to perform the subsequent data analysis such as QC, PCA, t-SNE, and other analyses. Only cells with mitochondrial read rate ≤10% and detectable genes ≥200 were retained for scRNA-seq analysis. The datasets of two cell samples from each diet group were integrated and analyzed together in the implementation of Seurat analysis. The scRNA-seq matrix count data were normalized, scaled and clustered using the top principal components of highly variable genes (HVGs). The t-SNE algorithm in the Seurat package was utilized to generate t-SNE plots and visualize gene features in the resulting cell clusters. Feature plots, Vlnplots, scatter plots and volcano plots for the identified cell clusters were generated after cell-cluster-specific markers were determined using the FindMarker algorithm in the Seurat package. The scRNA-seq raw and processed datasets have been deposited in the GEO data repository (GEO accession number: GSE152866).

### 4.4. Statistics and Reproducibility

ScRNA-seq was done on single-cell preparations from RD- and HFD-fed *C57BL6 Brca1−/−; p53+/−* mice (*n* = 2 for each group). The adjusted *p*-value < 0.05 was considered to be significant for differential gene expression analysis across two conditions.

## 5. Conclusions

In summary, our scRNA-seq study provides new mechanistic insight into how obesity promotes breast cancer development. Based on our findings, we hypothesize that obesity alters the mammary tissue microenvironment and immunity through deregulating stromal and immune cell functions. These obesity-mediated pathological effects create a pro-tumorigenic microenvironment to cultivate breast epithelial tumorigenesis (Figure 10). Given that our scRNA-seq study was conducted on the Brca1-deficiency mouse breast cancer model to elucidate the microenvironmental impact of obesity on breast cancer development, our findings may form the basis for the future development of obesity-related therapeutic and preventive interventions.

## DVA/US Government Disclaimer

The contents do not represent the views of the U.S. Department of Veterans Affairs or the United States Government.

## Supplementary Materials

The following are available online at https://www.mdpi.com/2072-6694/12/8/2235/s1, Figure S1: This QC report for the scRNA-seq of the RD-1 cell sample was generated from CellRanger analysis, Figure S2: This QC report for the scRNA-seq of the RD-2 cell sample was generated from CellRanger analysis, Figure S3: This QC report for the scRNA-seq of the HFD-1 cell sample was generated from CellRanger analysis, Figure S4: This QC report for the scRNA-seq of the HFD-2 cell sample was generated from CellRanger analysis, Figure S5: Gene enrichment analysis of biological function pathways in different stromal subtype cells. Three different classified pathways are shown here, including immune function pathways (A), receptor/kinase function pathways (B) and stress response pathways (C). The cut-off FDR (0.01, −log10 (FDR) = 2) is shown as a dash line in figures.
