# Supplementary material for: Single-Cell RNA-seq Reveals Obesity-Induced Alterations in the Brca1-Mutated Mammary Gland Microenvironment
Source: Cancers (Basel). 2020 Aug 10;12(8):2235. doi: 10.3390/cancers12082235 (PMC7464292; doi:10.3390/cancers12082235)
Supplement: Supplementary file 1 [file cancers-12-02235-s001.pdf]

Supplementary Materials

RD-1

Estimated Number of Cells

1,138

Mean Reads per Cell	Median Genes per Cell
72,123	1,604

Sequencing

Number of Reads	82,076,605
Valid Barcodes	98.2%
Sequencing Saturation	87.6%
Q30 Bases in Barcode	98.5%
Q30 Bases in RNA Read	76.7%
Q30 Bases in Sample Index	93.2%
Q30 Bases in UMI	98.7%

Mapping

Reads Mapped to Genome	92.6%
Reads Mapped Confidently to Genome	86.9%
Reads Mapped Confidently to Intergenic Regions	3.1%
Reads Mapped Confidently to Intronic Regions	11.1%
Reads Mapped Confidently to Exonic Regions	72.7%
Reads Mapped Confidently to Transcriptome	71.0%
Reads Mapped Antisense to Gene	0.7%

Cells

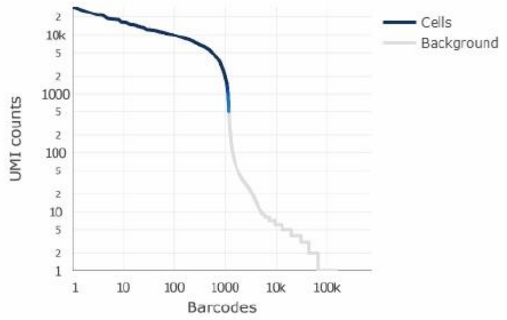

Estimated Number of Cells	1,138
Fraction Reads in Cells	96.0%
Mean Reads per Cell	72,123
Median Genes per Cell	1,604
Total Genes Detected	15,745
Median UMI Counts per Cell	4,843

Sample

Name	Sample_IL100119722
Description	
Transcriptome	mm10
Chemistry	Single Cell 3' v2
Cell Ranger Version	3.0.2

Figure S1. This QC report for the scRNA-seq of the RD-1 cell sample was generated from CellRanger analysis.

RD-2

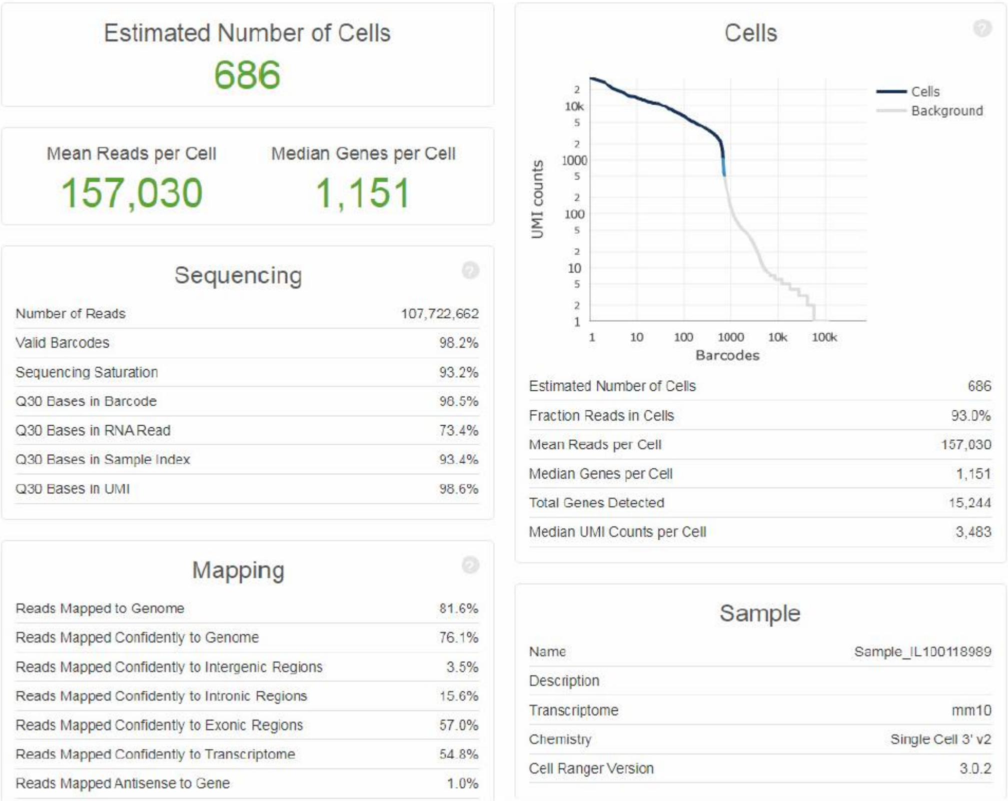

Estimated Number of Cells

686

Mean Reads per Cell	Median Genes per Cell
157,030	1,151

Sequencing

Number of Reads	107,722,662
Valid Barcodes	98.2%
Sequencing Saturation	93.2%
Q30 Bases in Barcode	98.5%
Q30 Bases in RNA Read	73.4%
Q30 Bases in Sample Index	93.4%
Q30 Bases in UMI	98.6%

Mapping

Reads Mapped to Genome	81.6%
Reads Mapped Confidently to Genome	76.1%
Reads Mapped Confidently to Intergenic Regions	3.5%
Reads Mapped Confidently to Intronic Regions	15.6%
Reads Mapped Confidently to Exonic Regions	57.0%
Reads Mapped Confidently to Transcriptome	54.8%
Reads Mapped Antisense to Gene	1.0%

Cells

Estimated Number of Cells	686
Fraction Reads in Cells	93.0%
Mean Reads per Cell	157,030
Median Genes per Cell	1,151
Total Genes Detected	15,244
Median UMI Counts per Cell	3,483

Sample

Name	Sample_IL100118989
Description	
Transcriptome	mm10
Chemistry	Single Cell 3' v2
Cell Ranger Version	3.0.2

Figure S2. This QC report for the scRNA-seq of the RD-2 cell sample was generated from CellRanger analysis.

HFD-1

Estimated Number of Cells

1,165

Mean Reads per Cell	Median Genes per Cell
71,641	1,754

Sequencing

Number of Reads	83,462,320
Valid Barcodes	98.2%
Sequencing Saturation	86.1%
Q30 Bases in Barcode	98.5%
Q30 Bases in RNA Read	78.4%
Q30 Bases in Sample Index	94.5%
Q30 Bases in UMI	98.6%

Mapping

Reads Mapped to Genome	93.0%
Reads Mapped Confidently to Genome	87.4%
Reads Mapped Confidently to Intergenic Regions	2.9%
Reads Mapped Confidently to Intronic Regions	11.7%
Reads Mapped Confidently to Exonic Regions	72.9%
Reads Mapped Confidently to Transcriptome	71.0%
Reads Mapped Antisense to Gene	0.8%

Cells

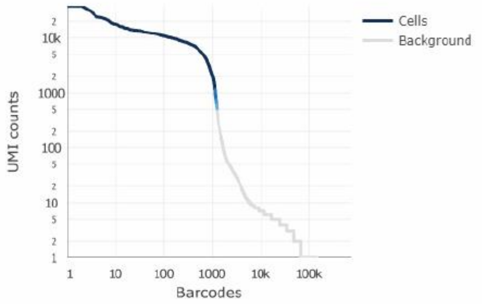

Estimated Number of Cells	1,165
Fraction Reads in Cells	95.0%
Mean Reads per Cell	71,641
Median Genes per Cell	1,754
Total Genes Detected	15,882
Median UMI Counts per Cell	5,595

Sample

Name	Sample_IL100119723
Description	
Transcriptome	mm10
Chemistry	Single Cell 3' v2
Cell Ranger Version	3.0.2

Figure S3. This QC report for the scRNA-seq of the HFD-1 cell sample was generated from CellRanger analysis.

HFD-2

Estimated Number of Cells

903

Mean Reads per Cell	Median Genes per Cell
99,942	1,459

Sequencing

Number of Reads	90,247,918
Valid Barcodes	98.2%
Sequencing Saturation	89.6%
Q30 Bases in Barcode	98.5%
Q30 Bases in RNA Read	74.6%
Q30 Bases in Sample Index	94.1%
Q30 Bases in UMI	98.7%

Mapping

Reads Mapped to Genome	90.9%
Reads Mapped Confidently to Genome	85.4%
Reads Mapped Confidently to Intergenic Regions	2.9%
Reads Mapped Confidently to Intronic Regions	13.1%
Reads Mapped Confidently to Exonic Regions	69.4%
Reads Mapped Confidently to Transcriptome	67.4%
Reads Mapped Antisense to Gene	0.8%

Cells

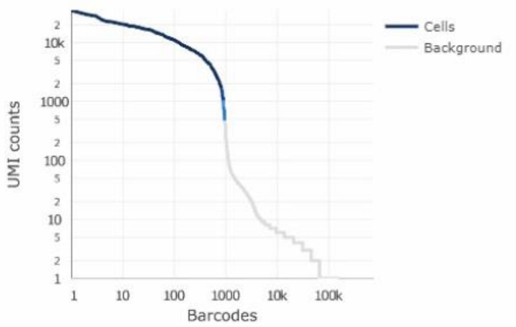

Estimated Number of Cells	903
Fraction Reads in Cells	94.9%
Mean Reads per Cell	99,942
Median Genes per Cell	1,459
Total Genes Detected	15,339
Median UMI Counts per Cell	4,586

Sample

Name	Sample_IL100118990
Description	
Transcriptome	mm10
Chemistry	Single Cell 3' v2
Cell Ranger Version	3.0.2

Figure S4. This QC report for the scRNA-seq of the HFD-2 cell sample was generated from CellRanger analysis.

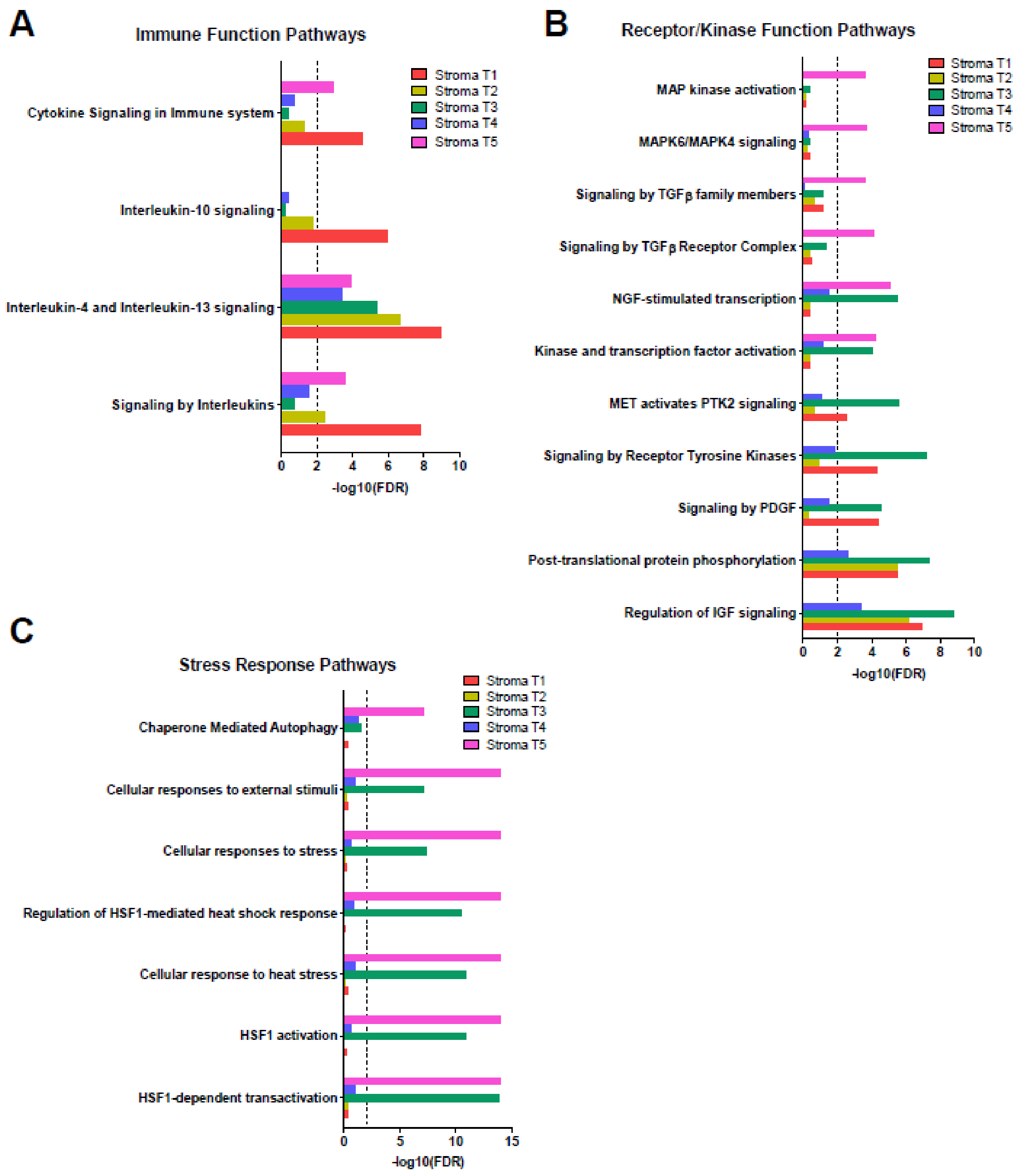

Figure S5. Gene enrichment analysis of biological function pathways in different stromal subtype cells. Three different classified pathways are shown here, including immune function pathways (**A**), receptor/kinase function pathways (**B**) and stress response pathways (**C**). The cut-off FDR (0.01, −log10(FDR) = 2) is shown as a dash line in figures.
